# Mental states in caregivers toward people with Alzheimer’s disease at different stages

**DOI:** 10.3389/fneur.2023.1327487

**Published:** 2024-01-11

**Authors:** Bei Li, Haiqiang Jin, Guiying Yan, Chen Zhang, Siwei Chen, Yue Wang, Ting Wang, Qiaoqin Wan, Zhimin Wei, Yongan Sun

**Affiliations:** ^1^Department of Neurology, Peking University First Hospital, Peking University, Beijing, China; ^2^Academy of Mathematics and Systems Science, Chinese Academy of Sciences, Beijing, China; ^3^School of Mathematical Sciences, University of Chinese Academy of Sciences, Beijing, China; ^4^The National Clinical Research Center for Mental Disorders and Beijing Key Laboratory of Mental Disorders, Beijing Anding Hospital, Capital Medical University, Beijing, China; ^5^Advanced Innovation Center for Human Brain Protection, Capital Medical University, Beijing, China; ^6^Nursing School of Peking University, Beijing, China; ^7^Health Service Department of the Guard Bureau of the Joint Staff Department, Beijing, China

**Keywords:** dementia, Alzheimer’s, caregivers, mental states, influencing factors, severity of disease

## Abstract

**Introduction:**

Caring for people with Alzheimer’s disease (AD) is burdensome, especially when family members act as caregivers. This multicenter survey first aimed to investigate caregivers’ mental states as well as its influencing factors in caring for people with different severities of AD in China.

**Methods:**

People with AD and their caregivers from 30 provincial regions in mainland China were enrolled from October 2020 to December 2020 to be surveyed for caregivers’ mental states and living conditions, as well as caregivers’ attitudes toward treatment and caring. Logistic regression was used to explore the factors that influence the positive and negative states of caregivers who care for people with different stages of AD.

**Results:**

A total of 1,966 valid questionnaires were analyzed (mild AD: 795, moderate AD: 521, severe AD: 650). A total of 73.6% of caregivers maintained normal states (mild group: 71.9%, moderate group: 73.9%, severe group: 75.2%; *X*^2^ = 2.023, *p* = 0.364), and the proportions of caregivers with positive and negative states were 26.3% (mild group: 38.4%, moderate group: 24.6%, severe group: 13.1%; *X*^2^ = 119.000, *p* < 0.001) and 36.5% (mild group: 25.2%, moderate group: 36.9%, severe group: 50.2%; *X*^2^ = 96.417, *p* < 0.001), respectively. The major factors that both influenced caregivers’ positive and negative states were the severity of AD, perceived efficacy of treatment, safety issues after AD dementia diagnosis and perceived social support (*p* < 0.005), while neuropsychiatric symptoms causing stress in caregivers (*p* < 0.001) only affected the negative states of caregivers. The results of further analysis according to disease severity showed that safety issues after AD dementia diagnosis (*p* < 0.005) only made significant differences in the mild-to-moderate group.

**Conclusion:**

To reduce negative states and promote positive states among caregivers, flexible and sensitive caregiving support could be built on caregivers’ demands in caring for people with different stages of AD. The support of emotion, social functioning and nursing skills is one of the significant ways for health workers to enhance caregivers’ competency.

## Introduction

Dementia is the most prevalent condition that necessitates caregivers in the aging population (1). The number of individuals globally with Alzheimer’s disease (AD), which is the major type of dementia, will reach 150 million by 2050 (2). According to the report of the World Health Organization (WHO) in 2019, dementia costs globally 1.3 trillion US dollars, and informal caregivers of family members with dementia provide on average 5 h of care and supervision per day (1). It is well known that dementia caregivers have more mental health problems than noncaregivers (3, 4). High levels of anxiety, depression, stress, and distress in caregivers of people with AD have been confirmed (5–8). In addition, the psychological burden may further cause somatic symptoms, such as hypertension and sleep disorders (5, 6). At the same time, mental burden could harm dyadic relationship quality and lead to caregivers’ negligence in mutual communication with people with AD (9). It could aggravate the difficulty of care when caregivers do not understand the need and the reason for psychobehavioral symptoms presented in patients (10). Therefore, efforts could be made to improve the mental health of caregivers.

Caregivers might express different mental states toward the demands of caring for people with AD. Indeed, the adaptation process of caregiving is characterized by the coexistence of both positive and negative experiences (11). In the negative aspect of caring, previous literature showed that caring for someone with AD was related to feelings of burden, perceived stress, depression, anxiety, and loneliness, especially after the wave of the COVID-19 pandemic (3, 7). However, Rosa et al. found that caregivers with a high level of capacity for successful adaptation in the face of stressful demands of care reported fewer emotional problems (12). There was evidence that caring duties could also bring positive experiences such as a sense of personal accomplishment and gratification (9). Positive aspects refer to the extent to which the caregiving roles are experienced as inspiring and rewarding, yielding positive consequences and enriching one’s lived experience (9, 13). Hence, the formulation of intervention strategies to reduce stress and optimize positive experiences are essential to maintain caregivers’ mental health.

The caregiving support services for AD can be developed on the basis of factors affecting both the positive and negative states of caregivers. In the transactional model of stress and coping, the evaluation of a positive or negative attitude toward a stressor depends on a secondary appraisal of whether one’s resources, abilities, and methods are adequate (9, 14, 15). According to stress models, a systematic review revealed that the important determinants of caregiver burden and depression were patients’ behavioral problems or mood disorders, and caregiver resources, for example personality traits, coping styles, and competencies, might be considered mediators (15). Another review integrated the findings regarding positive aspects of caregiving, and indicated that social support consisting of emotional support and social interactional support played an essential role in positivity (9). In addition, the care demands of people with various stages of AD are different, and the mental health of caregivers may be driven by different factors according to disease severity. In terms of information needs, caregivers of people with mild dementia needed information on pharmacotherapy of the disease, while those caring for people with moderate to severe dementia were primarily concerned about dementia prognosis and health care services (16). A previous study found that caregivers’ capacity for successful adaptation was influenced by different factors between the groups of mild and moderate AD (12). The increase of caregiver burden might be related to the severity of cognitive deficit and neuropsychiatric symptoms (17, 18). Nevertheless, the flexibility and sensitivity of services to the specific needs of caregivers are the key components of the successful adaptation for care of people with AD at different stages (16, 19). The factors affecting the positive and negative states of caregivers during disease progression might receive attention.

In China, there are almost 9.83 million people aged 60 years or older with AD (20). More than 60% of families in a previous study expressed a medium to high level burden of caring for people with AD (21). The process of stress and coping is affected by traditional Chinese culture, such as the concepts of family, and the mental state of these caregivers might gradually change as the patient’s condition changes (22, 23). There is insufficient evidence to explore the mental states of caregivers and form suitable and sensitive caregiving support systems in China. This national survey first aimed to investigate the caregivers’ states of positive and negative mental states as well as its influencing factors in caring for people with AD with different severities, and further offers basic insight for health workers and researchers in China and worldwide.

## Method

### Study design and samples

The process of this large-sample, multicenter, cross-sectional survey has been previously described (24). Participants from 30 provincial, municipal, and autonomous regions in mainland China were enrolled between October 2020 and December 2020. Patients were diagnosed with probable AD dementia by cognitive experts from comprehensive psychiatric hospitals through a series of tests according to the national diagnostic guidelines (25) and were considered potential participants. People with other neurological diseases that were related to cognitive impairment and major psychiatric disorders were excluded, and then patients or their caregivers were introduced to the purpose and content of this survey. If they agreed with and provided informed consent, caregivers completed the questionnaires.

### Measures and data collection

The demographic and clinical characteristics of people with AD were surveyed. This study also assessed caregivers’ mental states and living conditions, as well as caregivers’ attitudes toward treatment and caring. The questionnaire was derived from the results of a previous survey (21, 26) and had been modified by several specialists according to the aim of this study.

The assessments of demographic and clinical characteristics of people with AD were introduced previously (24), including gender, age, length of education, residential area, form of care, family earnings, and severity of AD. The severity of AD was estimated by neurologists based on patients’ symptoms and neuroimaging data according to the MTA criterion, which ranks the degree of hippocampal atrophy (27).

Caregivers’ mental states and living conditions were assessed with a self-designed questionnaire. Different feelings might coexist during the process of caregiving experience (9, 28). According to the valence of mental states (29), the item of caregivers’ current mental state toward patients was set as a multiple choice incorporating normal states, positive states and negative states. Normal states represented a willingness to bear hardships and fulfill responsibilities. Caregivers might passively drift along in this way. However, caregivers with positive states were full of hope and optimism. In contrast, negative states refers to a feeling of powerlessness, hopelessness, anxiety, or depression. Caregivers would be filled with despair and lose faith in the future. Caregivers’ living conditions had 7 options covering the possible changes in caregivers’ lives, such as the concept of value, relationship, time dependence, quality of life, and economic and emotional burden.

Caregivers’ attitudes toward treatment and caring were also assessed with self-designed items, including perceived efficacy of treatment, safety issues after AD dementia diagnosis, neuropsychiatric symptoms causing stress in caregivers, perceived social support, the situation of sharing troubles or stress, and the reasons for not talking about patients’ condition. The neuropsychiatric symptoms were derived from the Neuropsychiatric Inventory (NPI) (30). Considering the cooccurrence of specific symptoms in some individuals, making it difficult to determine the unique effect of each symptom, we classified these symptoms into three symptom clusters: hyperactivity, psychosis, and physical behavior symptom clusters (3, 31).

### Ethical consideration

This study was approved by the Ethics Committee of Peking University First Hospital. The right to informed consent, voluntary participation, and confidentiality of responses were verbally assured.

### Statistical analyses

All analyses were performed using SPSS 23.0 software. First, all variables were described as the means and SD or frequencies and percentages, as appropriate. Then, ANOVA and chi-square tests were used to calculate *p* values for differences, and further pairwise comparisons were performed to determine the differences between any two groups. Finally, binary logistic regression was used to explore the factors that influence the mental states of caregivers. On the basis of the mental states of caregivers, both positive and negative states were outcome variables. All the characteristics of patients and caregivers’ attitudes toward treatment and caring were considered potential influencing factors and filtered by the backward method with a likelihood ratio test. Among them, the multichoice variables were assigned as bivariate variables (e.g., whether or not safe issues have occurred after AD dementia diagnosis; whether or not there were gains from social support; whether or not sharing troubles or stress with others) to participate in regression analysis. In terms of the variable of neuropsychiatric symptoms, each symptom was assigned 1 point, and the total score of each participant was calculated for logistic analysis.

## Results

### Demographic and clinical characteristics of patients

A total of 2,813 questionnaires were collected, and 1,966 valid questionnaires were analyzed. Questionnaires were eliminated for the incorrect and incomplete filling of items. A total of 40.4% (*n* = 795) of patients had mild AD, 26.5% (*n* = 521) of patients had moderate AD, and 33.1% (*n* = 650) of patients had severe AD. The demographic and clinical characteristics of the groups are presented in Table 1. Their average age was 73.1 ± 10.7 years (mild: 70.8 ± 10.8; moderate: 74.3 ± 10.3; severe: 75.0 ± 10.3; *F* = 33.170, *p* < 0.001). The results of pairwise comparisons between the two groups showed that the moderate and severe groups were older than the mild group (*p* < 0.001). According to the results of univariate analysis, people with different severities of AD had significant variation in demographic and clinical variables. Most patients were male (57.6%), had less than 6 years of schooling (37.3%), lived in an urban area (72.6%), and were cared for by family (92.7%).

**Table 1:** The demographic and clinical characteristics of people with AD.

Variables	Severity of AD			Overall	*p* ^*^
	Mild	Moderate	Severe		
Overall	795 (40.4%)	521 (26.5%)	650 (33.1%)	1966 (100.0%)	
Gender					0.008
Male	363 (54.3%)	193 (63.0%)	278 (57.2%)	834 (57.6%)	
Female	432 (45.7%)	328 (37.0%)	372 (42.8%)	1,132 (42.4%)	
Age, years					<0.001
40–49	35 (4.4%)	6 (1.2%)	5 (0.8%)	46 (2.3%)	
50–59	76 (9.6%)	42 (8.1%)	45 (6.9%)	163 (8.3%)	
60–69	213 (26.8%)	110 (21.1%)	153 (23.5%)	476 (24.2%)	
70–79	295 (37.1%)	175 (33.6%)	207 (31.8%)	677 (34.4%)	
≥80	176 (22.1%)	188 (36.1%)	240 (36.9%)	604 (30.7%)	
Length of education					0.004
0–6 years	289 (36.4%)	230 (44.1%)	215 (33.1%)	734 (37.3%)	
7–9 years	169 (21.3%)	102 (19.6%)	126 (19.4%)	397 (20.2%)	
10–12 years	181 (22.8%)	99 (19.0%)	164 (25.2%)	444 (22.6%)	
≥13 years	156 (19.6%)	90 (17.3%)	145 (22.3%)	391 (19.9%)	
Residence					<0.001
Urban	514 (64.7%)	377 (72.4%)	536 (82.5%)	1,427 (72.6%)	
Rural	281 (35.5%)	144 (27.6%)	114 (17.5%)	539 (27.4%)	
Form of care					<0.001
Care at home	778 (97.9%)	481 (92.3%)	564 (86.6%)	1823 (92.7%)	
Care from professional institutions	17 (2.1%)	40 (7.7%)	86 (13.2%)	143 (7.3%)	
Family earning (monthly)						0.002
≤2000RMB	171 (21.5%)	124 (23.8%)	101 (15.5%)	396 (20.1%)	
2001 ~ 5000RMB	320 (40.3%)	198 (38.0%)	256 (39.4%)	774 (39.4%)	
5,001 ~ 8000RMB	153 (19.2%)	110 (21.1%)	131 (20.2%)	394 (20.0%)	
8,001 ~ 10000RMB	65 (8.2%)	45 (8.6%)	68 (10.5%)	178 (9.1%)	
10,001 ~ 15000RMB	39 (4.9%)	24 (4.6%)	56 (8.6%)	119 (6.1%)	
>15000RMB	47 (5.9%)	20 (3.8%)	38 (5.8%)	105 (5.3%)	

^*^p-values from Chi-square test.

### The caregivers’ mental states and living conditions

The results for caregivers’ mental states and living conditions across different stages from mild to severe AD are shown in Table 2. A total of 73.6% of caregivers were willing to bear hardships and fulfill responsibilities, and there were no significant differences across the three groups (X^2^ = 2.023, *p* = 0.364). While 36.5% of all caregivers and 50.2% of caregivers of people with severe AD expressed their negative mood, 26.3% of all caregivers and 38.4% of caregivers of people with mild AD felt a positive mood. Further analysis of pairwise comparison showed significant differences among all groups in the aspects of negative states (*X*^2^ = 96.417, *p* < 0.001) and positive states (*X*^2^ = 119.000, *p* < 0.001).

**Table 2:** The caregivers’ meatal states and living conditions.

Variables	Severity of AD			Overall	*p* ^*^
	Mild	Moderate	Severe		
Mental states of caregivers					
Positive states	305 (38.4%)	128 (24.6%)	85 (13.1%)	518 (26.3%)	<0.001
Normal states	572 (71.9%)	385 (73.9%)	489 (75.2%)	1,446 (73.6%)	0.364
Negative states	200 (25.2%)	192 (36.9%)	326 (50.2%)	718 (36.5%)	<0.001
Changes in life of caregivers					
Positive consequences in concept of value	517 (65.0%)	356 (68.3%)	434 (66.8%)	1,307 (66.5%)	0.455
Intimate connection with patients	369 (46.6%)	240 (46.1%)	224 (34.5%)	833 (42.4%)	<0.001
Without any changes	83 (10.4%)	34 (6.5%)	20 (3.1%)	137 (7.0%)	<0.001
Time dependence	326 (41.0%)	307 (58.9%)	456 (70.2%)	1,089 (55.4%)	<0.001
Increase of economic burden	243 (30.6%)	204 (39.2%)	333 (51.2%)	780 (39.7%)	<0.001
Decline of quality of life	207 (26.0%)	191 (36.7%)	327 (50.3%)	725 (36.9%)	<0.001
Increase of emotional burden	167 (21.0%)	161 (30.9%)	276 (42.5%)	604 (30.7%)	<0.001

^*^p-values from Chi-square test.

For changes in the life of caregivers, 66.5% showed an improvement in their concepts of value, and no significant differences were shown across the three groups (*X*^2^ = 1.573, *p* = 0.455). In terms of positive change, 34.5% of caregivers in the severe group indicated a closer relationship with the patients, and the severe group had a lower proportion than the mild or moderate group (*X*^2^ = 24.890, *p* < 0.001). However, 55.4% of all caregivers expressed a reduction in free time, and three groups showed significant differences (mild: 41.0%; moderate: 58.9%; severe: 70.2%; *X*^2^ = 126.540, *p* < 0.001). At the same time, among caregivers of people with severe AD, 51.2 and 50.3% of them expressed an increase in economic burden and a decline in quality of life, respectively. The results of further pairwise comparison indicated that their proportions were significantly different across the three groups (*p* < 0.001).

### Caregivers’ attitudes toward treatment and caring

The caregivers’ attitudes toward treatment and caring are shown in Table 3. A total of 75.7% did not perceive effective improvement from treatment, and 19.3% of them did not receive help in caring skills from social support. During the process of caring, the safety issues were falls (39.6%) and wandering away (37.5%). The neuropsychiatric symptoms causing stress in caregivers were physical behavior symptoms (78.3%). In the mild group, the safety issues were falls (25.9%) and taking the wrong medicine (24.0%), and the neuropsychiatric symptoms were hyperactivity symptoms that led to stress (72.2%). Moreover, 4.8% of caregivers did not share troubles or stress with others, because 19% of them believed it was useless except for comfort.

**Table 3:** Caregivers’ attitudes toward treatment and caring.

Variables	Severity of AD			Overall	*p* ^*^
	Mild	Moderate	Severe		
Perceived efficacy of treatment					<0.001
Improvement	276 (34.7%)	126 (24.2%)	94 (14.5%)	496 (25.2%)	
Stable condition	324 (40.8%)	152 (29.2%)	115 (17.7%)	591 (31.0%)	
Deterioration	195 (24.5%)	243 (46.6%)	441 (67.8%)	879 (44.7%)	
Safety issues after AD dementia diagnosis					
Never	444 (55.8%)	404 (77.5%)	559 (86.0%)	1,407 (71.6%)	<0.001
Falls	206 (25.9%)	219 (42.0%)	353 (54.3%)	778 (39.6%)	<0.001
Wandering away	142 (17.9%)	244 (46.8%)	352 (54.2%)	738 (37.5%)	<0.001
Taking the wrong medicine	191 (24.0%)	128 (24.6%)	107 (16.5%)	426 (21.7%)	<0.001
Pressure sores	20 (2.5%)	26 (5.0%)	103 (15.8%)	149 (7.6%)	<0.001
Appetite disorder	51 (6.4%)	59 (11.3%)	129 (19.8%)	239 (12.2%)	<0.001
Trauma	49 (6.2%)	27 (5.2%)	53 (8.2%)	129 (6.6%)	0.105
Neuropsychiatric symptoms causing stress in caregivers					
Physical behavior symptoms	555 (69.8%)	421 (80.8%)	564 (86.8%)	1,540 (78.3%)	<0.001
Hyperactivity symptoms	578 (72.2%)	392 (75.2%)	453 (69.7%)	1,423 (72.4%)	0.104
Psychosis symptoms	527 (66.3%)	345 (66.2%)	329 (50.6%)	1,201 (61.1%)	<0.001
Perceived social support					
Methods to maintain patients’ basic activity of daily living	514 (64.7%)	320 (61.4%)	378 (58.2%)	1,212 (61.6%)	0.041
Solutions to neuropsychiatric symptoms	436 (54.8%)	283 (54.3%)	346 (53.2%)	1,065 (54.2%)	0.827
Methods to care for daily activities of patients	377 (47.4%)	253 (48.6%)	321 (49.4%)	951 (48.4%)	0.755
Methods of entertainment or reminiscence	407 (51.2%)	249 (47.8%)	259 (39.8%)	915 (46.5%)	<0.001
Methods to prevent patient loss or accidental injury	355 (44.7%)	243 (46.6%)	299 (46.0%)	897 (45.6%)	0.757
Methods to care for basic living of patients	257 (32.3%)	187 (35.9%)	318 (48.9%)	762 (38.8%)	<0.001
Without effect	114 (14.3%)	113 (21.7%)	152 (23.4%)	379 (19.3%)	<0.001
The situation of sharing troubles or stress						0.023
Sometimes taking the initiative	260 (32.7%)	175 (33.6%)	228 (35.1%)	663 (33.7%)	
Only sharing with close families or friends	333 (41.9%)	231 (44.3%)	239 (36.8%)	803 (40.8%)	
Do not bring up the subject unless asked	155 (19.5%)	94 (18.0%)	157 (24.2%)	406 (20.7%)	
Never sharing	47 (5.9%)	21 (4.0%)	26 (4.0%)	94 (4.8%)	
Reasons for not talking about patients’ condition						0.045
Uselessness except for comfort	141 (17.7%)	85 (16.3%)	147 (22.6%)	373 (19.0%)	
Unwilling to disturb others	38 (4.8%)	16 (3.1%)	23 (3.5%)	77 (3.9%)	
Feeling ashamed	9 (1.1%)	5 (1.0%)	9 (1.4%)	23 (1.2%)	
Others	14 (1.8%)	9 (1.7%)	4 (0.6%)	27 (1.4%)	

^*^p-values from Chi-square test.

### Factors that influence the mental states of caregivers

The screening results of binary logistic regression exploring the relevant factors influencing the positive states of caregivers are shown in Table 4. The major factors that influence caregivers’ positive states were residence (*p* = 0.009, OR = 0.70, 95% CI: 0.54–0.91), severity of AD (*p* < 0.001, OR = 0.67, 95% CI: 0.58–0.77), perceived efficacy of treatment (*p* < 0.001, OR = 1.97, 95% CI: 1.70–2.28), safety issues after AD dementia diagnosis (*p* = 0.009, OR = 0.73, 95% CI: 0.57–0.92), and perceived social support (*p* = 0.001, OR = 1.77, 95% CI: 1.27–2.46). Further analyses with factors that influenced the positive states of caregivers were conducted in different stages of AD and found that perceived efficacy of treatment (*p* < 0.001) was the common factor.

**Table 4:** Binary logistic regressions of positive states of caregivers among different stages of AD.

Models	Variables	*p*	OR (95% CI)	Chi-square	*p*
All patients	Residence	0.009	0.70 (0.54, 0.91)	284.702	<0.001
	Severity of AD	<0.001	0.67 (0.58, 0.77)		
	Perceived efficacy of treatment	<0.001	1.97 (1.70, 2.28)		
	Safety issues after AD dementia diagnosis	0.009	0.73 (0.57, 0.92)		
	Perceived social support	0.001	1.77 (1.27, 2.46)		
Mild AD	Perceived efficacy of treatment	<0.001	1.94 (1.56, 2.43)	97.901	<0.001
	Safety issues after AD dementia diagnosis	0.017	0.68 (0.50, 0.94)		
	Perceived social support	<0.001	2.98 (1.72, 5.16)		
Moderate AD	Perceived efficacy of treatment	<0.001	2.05 (1.57, 2.69)	44.409	<0.001
Severe AD	Residence	0.005	0.43 (0.24, 0.78)	38.365	<0.001
	Perceived efficacy of treatment	<0.001	1.84 (1.38, 2.45)		

In all analyses, caregivers without positive states is the reference category.

The screening results of binary logistic regression exploring the relevant factors influencing the negative states of caregivers are shown in Table 5. Age (*p* < 0.001, OR = 0.82, 95% CI: 0.74–0.90), form of care (*p* < 0.001, OR = 1.99, 95% CI: 1.35–2.92), severity of AD (*p* < 0.001, OR = 1.40, 95% CI: 1.23–1.60), perceived efficacy of treatment (*p* < 0.001, OR = 0.65, 95% CI: 0.56–0.75), neuropsychiatric symptoms causing stress in caregivers (*p* < 0.001, OR = 1.17, 95% CI: 1.13–1.22), safety issues after AD dementia diagnosis (*p* = 0.004, OR = 1.43, 95% CI: 1.12–1.83), and perceived social support (*p* = 0.004, OR = 0.44, 95% CI: 0.34–0.56) entered the final model among all caregivers. The results of further analyses with factors that influenced the negative states of caregivers among different stages of AD showed that form of care (*p* > 0.05) was not significant in the mild group, while safety issues after AD dementia diagnosis (*p* > 0.05) was not significant in the severe group.

**Table 5:** Binary logistic regressions of negative states of caregivers among different stages of AD.

Models	Variables	*p*	OR (95% CI)	Chi-square	*p*
All patients	Age	<0.001	0.82 (0.74, 0.90)	347.174	<0.001
	Form of care	<0.001	1.99 (1.35, 2.92)		
	Severity of AD	<0.001	1.40 (1.23, 1.60)		
	Perceived efficacy of treatment	<0.001	0.65 (0.56, 0.75)		
	Neuropsychiatric symptoms causing stress in caregivers	<0.001	1.17 (1.13, 1.22)		
	Safety issues after AD dementia diagnosis	0.004	1.43 (1.12, 1.83)		
	Perceived social support	<0.001	0.44 (0.34, 0.56)		
Mild AD	Age	0.003	0.77 (0.65, 0.91)	93.596	<0.001
	Perceived efficacy of treatment	<0.001	0.63 (0.50, 0.80)		
	Neuropsychiatric symptoms causing stress in caregivers	<0.001	1.15 (1.09, 1.22)		
	Safety issues after AD dementia diagnosis	0.034	1.47 (1.03, 2.11)		
	Perceived social support	<0.001	0.37 (0.24, 0.58)		
Moderate AD	Age	0.047	0.82 (0.67, 1.00)	84.441	<0.001
	Form of care	0.037	2.17 (1.05, 4.51)		
	Perceived efficacy of treatment	<0.001	0.57 (0.44, 0.74)		
	Neuropsychiatric symptoms causing stress in caregivers	<0.001	1.14 (1.07, 1.22)		
	Safety issues after AD dementia diagnosis	0.011	1.94 (1.16, 3.22)		
	Perceived social support	<0.001	0.37 (0.23, 0.59)		
Severe AD	Form of care	0.015	1.90 (1.13, 3.18)	91.33	<0.001
	Perceived efficacy of treatment	0.010	0.73 (0.58, 0.93)		
	Neuropsychiatric symptoms causing stress in caregivers	<0.001	1.24 (1.16, 1.33)		
	Perceived social support	0.004	0.56 (0.37, 0.83)		

In all analyses, caregivers without negative states is the reference category.

## Discussion

AD is the leading cause of disability and dependency among elderly individuals, and the support of mental health for their carers has been underscored (3, 32). This national cross-sectional survey explored the situation of caregivers’ mental states as well as its influencing factors in caring for people with AD with different severities in China. These results hinted at distinctions in both the positive and negative states of caregivers during different stages of AD and further laid the foundation for health workers to provide flexible and sensitive caregiving support services.

Alzheimer’s Disease International (ADI) reported that over 50% of carers globally say their health has suffered as a result of their caring responsibilities even while expressing positive sentiments about their role (33). This proportion of caregivers with negative states was similar to our results in the severe group, while 26.3 and 36.5% of all caregivers had positive and negative states, respectively. Consistent with previous studies (28, 32, 34), time dependency, economic burden and decline of quality life were caregivers’ major predicaments in this study. Therefore, it is urgent to take targeted strategies to promote the positive states of caregivers and reduce their negative states, especially for caregivers of people with severe AD. When caregivers undertake caring responsibilities in different stages of the disease, it is essential to target caregivers’ needs for the provision of flexible and sensitive support (9, 34, 35).

The positive or negative states of caregivers depended on whether the carers could fulfill caregiving demands. Consistent with previous studies (9, 12, 15, 36), the regression analysis of positive and negative states in this study found that the common influencing factors were the severity of AD and perceived social support. Furthermore, the results of this survey detected that the efficacy of treatment and safety issues after AD dementia diagnosis would affect caregivers’ mental status, but the latter made significant differences in the mild-to-moderate group after stratified analysis according to the severity of AD. The efficacy of treatment affects the degree of self-care ability, which is related to the burden of caring tasks in the different stages of AD (3, 37). According to the results of this study, the common security issues were falls, getting lost, and taking the wrong medicine, which might be attributed to the symptoms of AD; however, less than half of caregivers in our survey mastered the methods to prevent accidental injury. The imbalance between care demands and competence promotes negative states and reduces positive states (9, 15). Social and instrumental support, including emotion, social functioning, and nursing skills, could mediate the effects of caregivers’ stressors and consequently arouse distinct mental reactions of caregivers (12, 38). In short, it is worth noting that effective treatment to maintain patients’ activities of daily living and caregiving support from others were essential strategies for caregivers’ mental health. The skills of nursing safety could be reinforced especially among the caregivers of people with mild-to-moderate AD.

To develop targeted strategies for caregivers’ mental health, discrepancies in influencing factors between positive and negative states cannot be ignored. Regarding sociodemographic factors, this study showed that residence was related to caregivers’ positive states, especially in the severe group. More caregivers showed positive states in rural areas, which might be attributed to the patients in rural areas who more frequently participate in nonpharmaceutical therapy. As the previous article described, these nonpharmaceutical activities, including physical activity, mental activity (e.g., chess, mahjong), and musical therapy, were demonstrated to be beneficial for the improvement of cognition and neuropsychiatric symptoms (24). On the other hand, caregivers could receive support from health workers and have time for respite during the patients’ participation in nonpharmaceutical treatment. Moreover, in our survey, age and form of care correlated with caregivers’ negative states. Meanwhile, age affected caregivers’ mental states among mild-to-moderate groups, while the form of care influenced their states in moderate-to-severe groups. If patients are older, they might be more dependent, and their caregivers spend more time in daily caring (3, 34). Caregivers more frequently experienced negative states when patients lived in professional institutions, which might be explained by the regional cultural characteristics in China. Traditional cultural heritage, referred to as filial piety, made caregivers feel guilt, as they abandoned their families to nursing homes (6, 21). Guilt presents obstacles for caregivers in acknowledging their value in protecting and maintaining the patient’s wellbeing (6). Thus, to promote positive states, caregivers could facilitate patients’ participation in nonpharmaceutical activities that are conducive to delaying disease progression. However, caregivers of people with moderate-to-severe AD or a lack of self-care ability could ask for professional help when they cannot afford the caring responsibilities and experience negative states. For instance, respite care services and nursing homes are alternative sources for both patients’ and caregivers’ health.

In addition, it is important to note that neuropsychiatric symptoms were only concerned with caregivers’ negative states in this study, regardless of the severity of AD. Previous reviews also agree that patients’ neuropsychiatric symptoms were the main detector of caregiver burden (15, 37). Even though a question remains as to whether certain symptoms are more challenging than others and are more useful indicators of the caregiver’s need for assistance or intervention, the presence of different neuropsychiatric symptoms in patients carries different distress patterns in caregivers (3, 8, 18). This study showed that the main symptoms causing caregiver stress were hyperactivity symptoms in the mild group and physical behavior symptoms in the moderate-to-severe group. A systematic review found that the application of problem-focused and emotion-focused coping strategies could ameliorate caregivers’ negative emotions (39). Jung et al. (10) identified the conceptual model of caregiver competence in managing neuropsychiatric symptoms, which laid the foundation for future education or training programs. In brief, the skills and competence of coping with neuropsychiatric symptoms could be grasped by caregivers for relief from negative states when facing patients’ demands at different stages.

This study was a national survey to explore the status of caregivers’ mental states and its influencing factors among people with different stages of AD. However, the study is not without its limitations. First, we cannot exclude selection bias because only caregivers who had access to our recruitment channels could participate in the survey, and there was a gap between the number of participants recruited in urban and rural areas. Nevertheless, the study population had a broad sociodemographic spectrum, which provides support at least to some extent, for the external validity of our results. Second, recall bias could hardly be excluded, which might affect the reported experiences of caring. Third, this survey adopted self-designed questionnaires that originated from the results of a previous survey and were modified by several specialists. Although the results had restrictions in comparability with studies from other countries, the mental states of carers in China was revealed to pave the way for future management of mental health.

## Conclusion

This study in China is the first to feature a large sample to explore the caregivers’ mental states and its influencing factors. To reduce their negative states and promote positive states, flexible and sensitive caregiving support could be built on caregivers’ demands in caring for people with different stages of AD. During the stage of mild-to-moderate AD, caregivers’ skills in nursing safety might be strengthened. When caring for people with moderate-to-severe AD, nonpharmaceutical treatment was regarded as a compensatory method of pharmacotherapy to relieve patients’ neuropsychiatric symptoms, and it was an alternative to receiving institutional care with professional help. In addition, it is important to know that the skills and competence of coping with neuropsychiatric symptoms in all stages of AD are alleviators of the negative states of caregivers.

## Data availability statement

The original contributions presented in the study are included in the article/supplementary material, further inquiries can be directed to the corresponding authors.

## Ethics statement

The studies involving humans were approved by the Ethics Committee of Peking University First Hospital. The studies were conducted in accordance with the local legislation and institutional requirements. The participants provided their written informed consent to participate in this study.

## Author contributions

BL: Writing – original draft, Writing – review & editing. HJ: Writing – original draft, Writing – review & editing. GY: Formal analysis, Writing – review & editing. CZ: Formal analysis, Investigation, Writing – review & editing. SC: Data curation, Investigation, Writing – review & editing. YW: Supervision, Writing – review & editing. TW: Data curation, Investigation, Writing – review & editing. QW: Methodology, Writing – review & editing. ZW: Project administration, Supervision, Writing – review & editing. YS: Funding acquisition, Project administration, Resources, Supervision, Writing – review & editing.
